# Generativity Development Across Adulthood: A Longitudinal Investigation

**DOI:** 10.1093/geroni/igaa057.1496

**Published:** 2020-12-16

**Authors:** Niccole Nelson, Cindy Bergeman

**Affiliations:** University of Notre Dame, Notre Dame, Indiana, United States

## Abstract

The developmental trajectory of Generativity, or investment in the next generation, has been theorized about for decades. Although Generativity is widely hypothesized to peak in midlife, and thus, follow a nonlinear change trajectory across adulthood, extant studies have been too limited in scope to formally test this hypothesis. Indeed, most existing studies on Generativity development have been cross-sectional, with the few longitudinal studies either only examining the first half of adulthood or using too few measurement points. The current study, therefore, aimed to address these limitations by investigating Generativity development in the context of an accelerated longitudinal design. Accelerated longitudinal designs capitalize on both cross-sectional and longitudinal data, combining age-heterogenous individuals’ overlapping trajectories to estimate developmental change across the sample’s age range. If cohort effects are not present in the estimated trajectory, this trajectory can be interpreted as developmental change. Participants included 876 age-heterogenous individuals from The Notre Dame Study of Health & Well-being (Mean age = 58.89; SD age = 9.42), a 10-year, longitudinal study of adult development and aging. Capitalizing on the age-heterogeneity of the sample at Time 1, two-level, multilevel modeling was employed to estimate Generativity development across ages 37 to 96. Results indicate that Generativity follows an age-graded cubic trend, with no apparent cohort effects. Specifically, Generativity peaks in early midlife, declines slightly before stabilizing across ages 47-77, and then declines sharply thereafter. Implications for lifespan developmental research, as well as health and well-being, will be discussed.

